# The extent of the hip bone sexual dimorphism in two Italian coeval modern skeletal samples

**DOI:** 10.1038/s41598-025-86197-3

**Published:** 2025-01-19

**Authors:** Rita Sorrentino, Annalisa Pietrobelli, Davide Mameli, Teresa Nicolosi, Maria Giovanna Belcastro

**Affiliations:** 1 Department of Biological, Geological and Environmental Sciences, University of Bologna, Via Selmi 3, Bologna, 40126 Italy; 2 Department of Human Origins, Max Planck Institute for Evolutionary Anthropology, Deutscher Platz 6, 04103 Leipzig, Germany; 3 Department of Cultural Heritage, University of Bologna, Ravenna, Italy

**Keywords:** Coxal bone, Sexual dimorphism, Documented human osteological collections, Evolution, Anatomy

## Abstract

The rate of sexual dimorphism in the human hip bone is primarily due to the structural demands of childbirth. Genetic, environmental, and socio-cultural factors can also influence pelvic shape variations across populations. This study examines intra-population sex variation within the Italian population based on regional differences of 280 coxal bones from two documented human osteological collections (Bologna and Sassari) coming from different geographical areas, the northern continental and island regions. Nineteen metric variables were used to evaluate sexual dimorphism and population differences. Most of the variables showed sexual dimorphism, particularly the hip height and ischio-pubic measures within both populations, and accurately predicted sex for each population (Bologna: 100%; Sassari: 91.2%). Results show the Bologna sample have larger dimensions for most of the measurements than the Sassari one, especially when considering the longitudinal ones. Some female traits of the Bologna sample are larger than the correspondent ones in the Sassari males. The rate of sexual dimorphism between the populations shows significant differences, with better sex distinction for Bologna than Sassari. This study aims at interpreting these intra-population differences, considering the effect of environmental (physical and social milieu) and genetic factors, underscoring the importance of this local differences for accurate diagnostic criteria.

**Supplementary Information:**

The online version contains supplementary material available at 10.1038/s41598-025-86197-3.

## Introduction

The pelvis – composed of two hip bones, the sacrum, and the coccyx – is the most dimorphic element in the human skeleton. Although this specialization is also exhibited by other primates, humans display a higher magnitude of sexual dimorphism^1,2^. From an evolutionary perspective, the human pelvic shape is the result of integrated biomechanical adaptations for efficient bipedal walking, leading to an optimized anatomical structure that supports internal organs and meets the demands of childbirth^3–6^.

Anatomical differences that are specifically related to sexual dimorphism are manifested throughout the whole structure of the human pelvis, both in shape and size^7,8^. In general, the female pelvis has a wider and broader structure, expressed by a wider bispinous width, with less prominent ischial spines, while the male pelvis shows a wider and more curved sacrum and a narrower subpubic arch^9–11^. These features are related to an expanded birth canal in females with a straighter sacrum and a wider subpubic angle, as a result of structural modifications to ensure the passage of large-brained neonates^9,10,12^. In addition, the unique rotational childbirth mechanism, rooted into the evolutionary origins of our species, likely played a role in determining the shape patterns of pelvic dimorphism^2,12,13^.

There is still a debate on whether these dimorphic differences were due to such obstetric constraints or differential sex-specific allometric growth trajectories and hormone (i.e., androgens and estrogens) influence and susceptibility during the skeletal development^1,11,12,14–17^. More broadly, sexual dimorphism expressed in the human skeleton could be explained by the complex interaction of selective pressure forces affecting the genotype while interacting with environmental influences on the phenotype^11,17^. Factors such as body size, age, activity patterns, diet, and population-specific variation should also be considered^18^. Murail and coauthors^19^ stressed the role of population-specific sexual dimorphism on a modern world-wide sample of coxal bones. Also, Vacca and Di Vella^20^, analyzing a sample of hip bones of individuals with known sex from Apulia (Italy), reported discriminant functions for metric sex determination, pointing out the necessity of population-specific analysis. On the contrary, Steyn and Patriquin^21^ highlighted that very little of the accuracy in sex determination based on discriminant function formulae is due to population specificity, providing formulae that can be used for a variety of populations. Indeed, this approach based on quantitative techniques to assess sex, such as metric evaluation and multivariate discriminant analysis, derives from the concept that within mammals, dimorphism is particularly expressed in body size, being males larger than females (called sexual size dimorphism). This is typically attributed to sexual selection promoting larger male body size^11^. Furthermore, previous studies suggested that primates, including humans, are characterized by increasing dimorphism with increasing body mass. In contrast, lower dimorphism was observed in small-body mass mammals [^22–25^, although see ^11^].

Environmental factors (e.g., different geographic and climatic features, socio-cultural background) and genetics may also contribute to human sexual dimorphism, differentially shaping body and hip size and morphology in various populations. This interplay of factors posed some challenges on possible interpretations on the intra- and inter-population differences, but also different rate of sexual dimorphism^26^.

This study examines the sexual dimorphism in the metrics of the human hip bone of two contemporary Italian samples from Bologna and Sassari, part of a large modern (19th -20th c.) documented (for age-at-death, sex, and cause of death) human osteological collection (DHOC) housed at the University of Bologna^27,28^. In both samples, most individuals were born and died in their respective cities and the birth date spans from the early 19th century (1814) up to the first years of the 20th century (1922). Individuals belong to a medium-low social status, as inferred from their occupation-at-death^27^. Additionally, the lifespan of the individuals of the two samples coincides with the long and difficult historical process that led to the unification and proclamation of the Italian Kingdom (1861–1946). Geographically, the samples come from very different areas. Bologna is placed in northern and continental Italy, close to the Po Plain and the Apennine Mountains, at 54 m above sea level, and is characterized by a continental climate. Sassari is located on an island (Sardinia, the second biggest island of the Mediterranean Sea), at 225 m above sea level, on a karstic plateau surrounded by valleys and gorges. The climate is usually warm-temperate, typical of Mediterranean region^29^.

In addition, the stratified prehistoric and historical peopling dynamics have differently characterized the Italian mainland and islands^30,31^. Regarding the genetic signals, three main areas across the peninsula - southern, northern, and Sardinia - are observed. The first two groups (i.e., southern and northern Italy), coinciding with the mainland, are genetically closer to the populations originated from Western Europe (Paleolithic European hunter-gatherers) and Eastern/Central Europe (following the migratory flows of steppe human groups during the Bronze Age^32^). On the other hand, Sardinian genetic ancestry is still today closer to Middle Eastern/Anatolian human groups, referring to the Neolithic transition and then retained as a consequence of its isolation^33^. Genetic distances among those regions have also been expressed by significant linguistic diversity^34,35^. Finally, differences in body size and stature between Bologna and Sassari have been already observed, as the former is characterized by greater stature than the latter^36^, allowing to compare this phenomenon to the well-known ‘island rule’ observed in many faunas that show smaller body size^37^.

Thus, using a set of metrical variables, we aim at (1) exploring the hip bone sexual dimorphism within and between the Bologna and Sassari groups (2) and comparing the extent of sexual dimorphism between these samples. We expect to find differences in size and degree of sexual dimorphism considering the above-mentioned genetic, climatic, and socio-cultural backgrounds of these two populations.

## Results

**Fig. 1:**
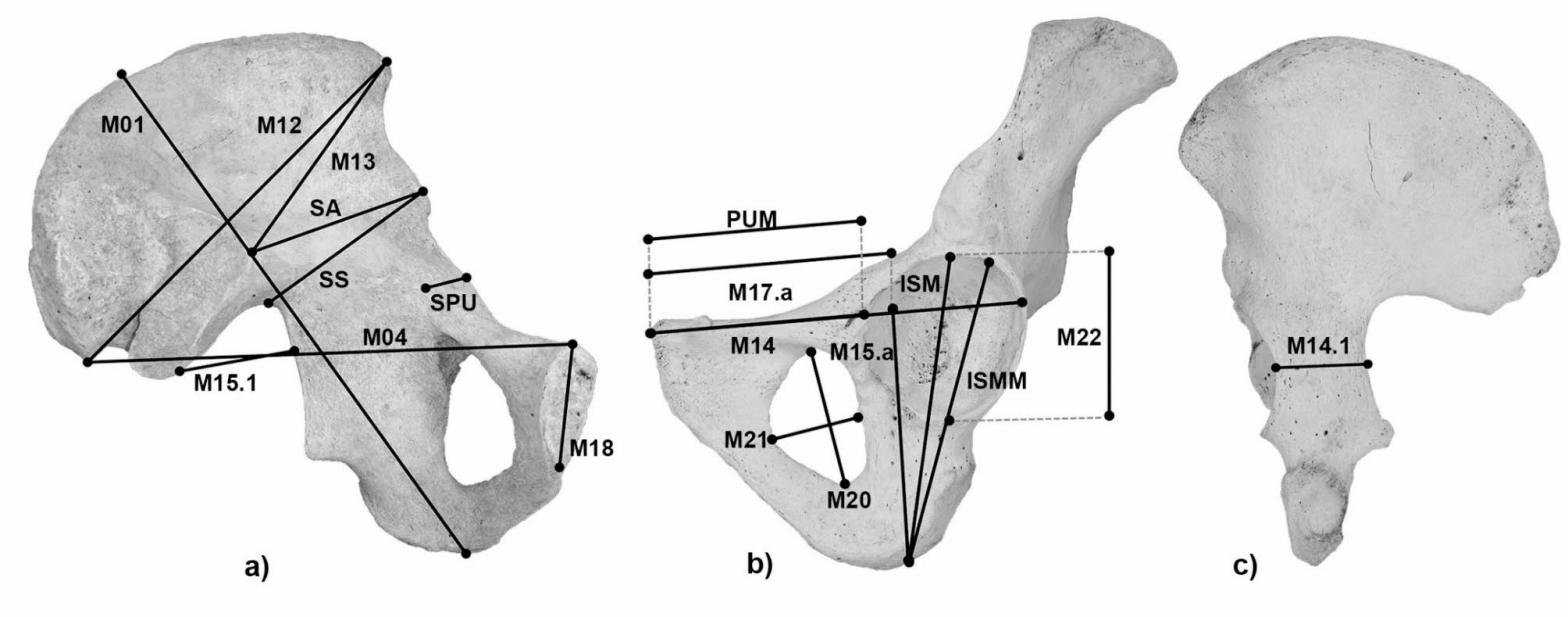
Left hip bone in medial (**a**), lateral (**b**), and posterior (**c**) views, with the representation of the 19 metrical variables used in this study.

This study considered a set of 19 hip metric variables (Table 1; Fig. 1) on a sample of 280 paired os coxae (140 left and 140 right) of adult individuals (> 20 years) from Bologna and Sassari collections (Table 2).

**Table 1:** Metric variables used in this study.

Measurement	Brief definition
M01^1^	Coxal bone maximum height: from the ischial tuberosity to the most superior point of the iliac crest
M04^1^	Coxal bone depth: from postero-superior iliac spine and superior margin of pubic symphysis
M12^1^	Iliac breadth, maximum: from the antero-superior to the postero-superior iliac spines
M13^1^	Breadth of iliac fossa: linear distance from antero-superior iliac spine to auricular surface margin
M14^1^	Acetabular – symphyseal breadth: from the superior-medial point of the pubic symphysis to the posterior margin of the acetabulum
M 14.1^1^	Cotylo-sciatic breadth: from the lateral border of the acetabulum to the midpoint of the anterior border of greater sciatic notch
M15.1^1^	Greater sciatic notch height: from the anterior border of the greater sciatic notch to the posteroinferior iliac spine (intersection between the auricular surface and the posterior border of greater sciatic notch)
M15.a^1^	Ischium length, acetabular: from the ischial tuberosity to the acetabular point
M17.a^1^	Pubic length, acetabular: from the superior point of the pubic symphysis to the acetabular point
M18^1^	Pubic symphysis height: from superior to inferior margins of pubic symphysis.
M20^1^	Obturator foramen length: from the most superior point of the superior border to the farthest point on the inferior border
M21^1^	Obturator foramen breadth: maximum distance from the posterior to the anterior border (perpendicular to the foramen length)
M22^1^	Maximum diameter of acetabulum: vertical diameter of acetabular rim
PUM^2^	Pubis length, modified: from the superior point of the pubic symphysis to the nearest acetabulum rim
ISM^2^	Ischium length, modified: from the ischial tuberosity to the most superior point of the acetabulum rim
SPU^2^	Cotylo-pubic breadth: from the most lateral acetabular point to medial border of the pubis (perpendicular to the axis of the pubis)
ISMM^2^	Ischium length, post-acetabular: from the most anterior–inferior point of the ischial tuberosity to the farthest point on the acetabular rim
SS^2^	Spino-sciatic length: from the antero-inferior iliac spine to the deepest point of the greater sciatic notch
SA^2^	Spino-auricular length: from the antero-inferior iliac spine to auricular point (intersection between arcuate line and auricular surface)

^1^Measurement code retrieved from Martin and Saller (1957).

^2^Measurement code retrieved from Vacca abd Di Vella (2012).

**Table 2:** The sample examined.

	Bologna		Sassari		
	M	F	M	F	Total
YA	13	16	8	16	53
MA	9	10	14	11	44
OA	14	8	14	7	43
Total	36	34	36	34	140

*YA* Young Adult (20–35 years), *MA* Middle Adult (36–50 years), *OA* Old Adult (> 50 years) (Buikstra & Ubelaker, 1994).

The results of the intra- and inter-observer error show high reliability for each metric variable with an Interclass correlation coefficient (ICC) value > 0.9 (Table 3). The paired t-test carried out to test the bilateral asymmetry shows no statistically significant differences between metric variables of right and left side, except for the cotylo-pubic breadth (SPU) (Table 4). Therefore, the following analysis considers only the right side. To test the normality assumption in the distribution of the data set variables, the Shapiro–Wilk tests show that metric variables are differently distributed depending on sex and population (Table S1), and the use of parametric or non-parametric tests in the following analysis is consistent with the distribution of the variables.

**Table 3:** Intraclass correlation coefficient (ICC) for intraobserver and interobserver errors.

Variables	Intraobserver		Interobserver	
	n	ICC	n	ICC
M01	37	0.998	10	0.998
M04	23	0.990	10	0.996
M12	30	0.983	10	0.970
M13	34	0.980	10	0.969
M14	28	0.970	10	0.975
M 14.1	39	0.996	10	0.927
M15.1	39	0.986	10	0.903
M15.a	38	0.993	10	0.978
M17.a	27	0.985	10	0.926
M18	26	0.975	10	0.921
M20	38	0.991	10	0.989
M21	35	0.955	10	0.936
M22	39	0.991	10	0.954
PUM	27	0.984	10	0.973
ISM	39	0.970	10	0.952
SPU	38	0.970	10	0.994
ISMM	38	0.977	10	0.976
SS	38	0.991	10	0.988
SA	38	0.991	10	0.989

**Table 4:** The paired t-test of comparison of left and right measurements.

Variables	Mean difference (L-*R*)	t value	*p* value
M01	0.477	0.316	0.752
M04	1.300	−0.846	0.398
M12	0.454	−0.405	0.685
M13	−0.729	1.021	0.307
M14	0.457	−0.507	0.612
M 14.1	−0.093	0.215	0.829
M15.1	−1.000	1.483	0.139
M15.a	−0.372	0.480	0.631
M17.a	0.0842	−0.114	0.909
M18	−0.123	0.196	0.844
M20	−0.181	0.337	0.736
M21	0.284	-0.756	0.449
M22	0.051	−0.095	0.924
PUM	−0.263	0.357	0.721
ISM	−0.750	0.792	0.428
SPU	−0.912	2.128	0.034
ISMM	0.007	−0.007	0.994
SS	−0.611	0.910	0.363
SA	−0.315	0.505	0.613

**Fig. 2:**
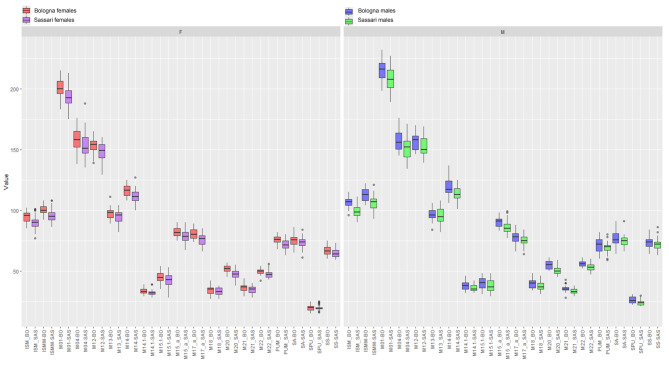
Box plots depicting distribution of coxal bone metrical variables in interaction between sex and population.

Table 5 presents the descriptive statistics (mean, standard deviation, and range) and the results of ANOVA or Kruskal Wallis for Sassari and Bologna sample. Most of the metric variables show significant differences between sexes in each sample, except for hip bone depth (M04), iliac breadth (M12), breadth of iliac fossa (M13), acetabular-symphyseal breadth (M14), and spino-auricolar length (SA) for both Bologna and Sassari. In addition, acetabular pubic length (M17.a) shows significant difference between sexes only in the Bologna sample, whereas obturator foramen breadth (M21) is significantly discriminant only between sexes of Sassari. For both Sassari and Bologna males and females, the highest standard deviations are found for hip maximum height (M01) and depth (M04) suggesting high degree of dispersion, while the lowest standard deviations are found for cotylo-sciatic breadth (M 14.1) and cotylo-pubic breadth (SPU). Although subtle asymmetry was detected for SPU (*p* = 0.034), the results show that both sides yield similar values (Table 5 and Table S1). The comparison between Bologna and Sassari (Table 6) shows significant differences between mean values of female and male groups respectively, as males and females of Bologna have mostly larger measurements than the Sassari ones (Fig. 2). It is also observed that various metrics are larger in the females of Bologna than in the males of Sassari (Table 5; Fig. 2).

**Table 5:** Descriptive statistics and results of ANOVA (F) or Kruskal Wallis (X^2^) for Sassari and Bologna.

Variables	Males				Females						
	n	Mean	SD^1^	Range	n	Mean	SD^1^	Range	*X* ^*2*^	*F*	*p* - value
Sassari											
M01	36	208.14	10.51	189–227	34	193.50	8.85	175–213	–	39.500	0.000
M04	30	150.93	8.99	134–171	25	153.16	11.91	135–188	–	0.623	0.433
M12	31	151.77	8.15	139–169	30	148.73	7.48	129–160	–	2.301	0.135
M13	36	95.47	6.24	82–108	34	94.82	5.35	82–104	–	0.217	0.643
M14	34	113.41	6.19	101–125	27	111.59	5.52	100–127	–	1.427	0.237
M 14.1	36	36.06	2.68	32–42	34	32.09	2.43	28–39	31.574	–	0.000
M15.1	35	38.26	4.91	29–48	34	42.85	5.94	28–53	–	12.280	0.000
M15.a	36	86.19	5.27	77–99	34	78.79	4.90	67–90	–	36.930	0.000
M17.a	34	75.26	4.58	64–85	26	76.15	4.79	66–85	–	0.534	0.468
M18	33	37.09	3.65	31–46	25	33.2	3.19	27–38	–	17.980	0.000
M20	35	50.57	3.32	45–59	34	47.65	3.79	38–55	–	11.650	0.000
M21	34	33.21	2.37	29–38	34	34.59	2.82	28–40	–	4.788	0.032
M22	36	53.22	3.15	47–60	34	47.68	3.35	43–56	30.211	–	0.000
PUM	34	69.26	4.51	59–80	26	71.77	4.74	63–80	–	4.346	0.041
ISM	36	99.17	5.53	90–111	34	89.91	5.60	77–101	–	48.370	0.000
SPU_dx	36	24.14	2.26	21–30	34	19.85	2.22	16–25	35.782	–	0.000
SPU_sx	37	25.32	2.99	20–33	35	20.94	2.40	16–26	–	46.660	0.000
ISMM	36	106.25	6.07	93–121	34	95.91	5.52	86–108	31.963		0.000
SS	36	71.50	4.75	63–86	33	64.58	3.89	59–73	–	43.440	0.000
SA	36	74.89	4.66	66–91	33	73.21	4.82	61–84	20.582	–	0.151
Bologna											
M01	33	214.82	8.74	198–232	33	200.06	7.57	183–215	–	53.750	0.000
M04	26	157.15	8.92	145–176	21	158.24	10.33	138–176	–	0.149	0.701
M12	25	156.08	6.87	146–170	29	153.66	6.12	139–165	–	1.883	0.176
M13	29	97.03	5.07	84–106	32	97.38	4.88	89–111	–	0.071	0.790
M14	31	118.48	7.16	106–137	22	116.05	4.87	108–125	–	1.914	0.173
M 14.1	35	38.1	3.55	32–46	34	33.3	2.47	29–39	–	41.58	0.000
M15.1	34	39.88	4.69	31–48	34	44.94	4.85	35–54	–	19.120	0.000
M15.a	35	90.06	4.43	83–98	34	81.82	3.91	75–90	–	65.920	0.000
M17.a	31	77.68	5.42	66–88	21	81.05	4.02	74–89	–	5.907	0.019
M18	29	39.48	4.01	34–48	22	33.68	4.13	27–42	–	25.460	0.000
M20	35	54.86	3.43	50–61	33	51.42	3.03	45–57	12.683	–	0.000
M21	35	35.54	2.65	28–43	30	36.23	3.14	29–44	11.863	–	0.276
M22	34	56.15	2.50	52–61	34	49.71	2.28	42–54	47.721	–	0.000
PUM	31	71.23	5.92	60–82	21	75.67	3.9	68–82	–	9.103	0.004
ISM	35	106.43	4.61	96–115	34	94.74	4.46	85–102	–	114.600	0.000
SPU_dx	34	25.97	2.75	22–31	34	19.82	2.35	15–25	42.847	–	0.000
SPU_sx	33	26.42	2.55	22–34	33	21.06	2.50	17–28	36.604	–	0.000
ISMM	35	112.69	5.05	104–122	34	100.68	4.00	92–108	–	119.6	0.000
SS	34	73.41	4.49	64–84	34	66.79	3.73	60–75	–	43.720	0.000
SA	34	76.12	6.14	64–91	34	75.12	4.82	65–86	–	0.558	0.458

^1^Standard deviation.

**Table 6:** ANOVA (F) or Kruskal Wallis (X^2^) comparison within sexes between populations.

Variables	Males			Females		
	Bologna vs. Sassari			Bologna vs. Sassari		
	*X* ^*2*^	*F*	*p* - value	*X* ^*2*^	*F*	*p* - value
M01	–	8.154	0.006	–	10.604	0.002
M04	–	6.710	0.012	–	2.340	0.133
M12	–	4.433	0.040	–	7.625	0.008
M13	–	1.186	0.280	–	4.082	0.048
M14	–	9.366	0.003	–	8.747	0.005
M 14.1	6.099	-	0.013	5.103	–	0.023
M15.1	–	1.974	0.165	–	2.519	0.117
M15.a	–	11.157	0.001	–	8.250	0.005
M17.a	–	3.780	0.056	–	13.968	0.001
M18	–	6.035	0.017	–	0.203	0.655
M20	20.445	–	0.000	–	20.226	0.000
M21	13.739	–	0.000	–	4.883	0.031
M22	–	18.411	0.000	10.982	–	0.000
PUM	–	2.279	0.136	–	9.177	0.004
ISM	–	36.003	0.000	–	15.434	0.000
SPU	7.362	–	0.006	0.068	–	0.794
ISMM	–	23.542	0.000	15.917		0.000
SS	–	2.990	0.088	–	5.677	0.020
SA	1.086	–	0.297	–	2.616	0.111

**Fig. 3:**
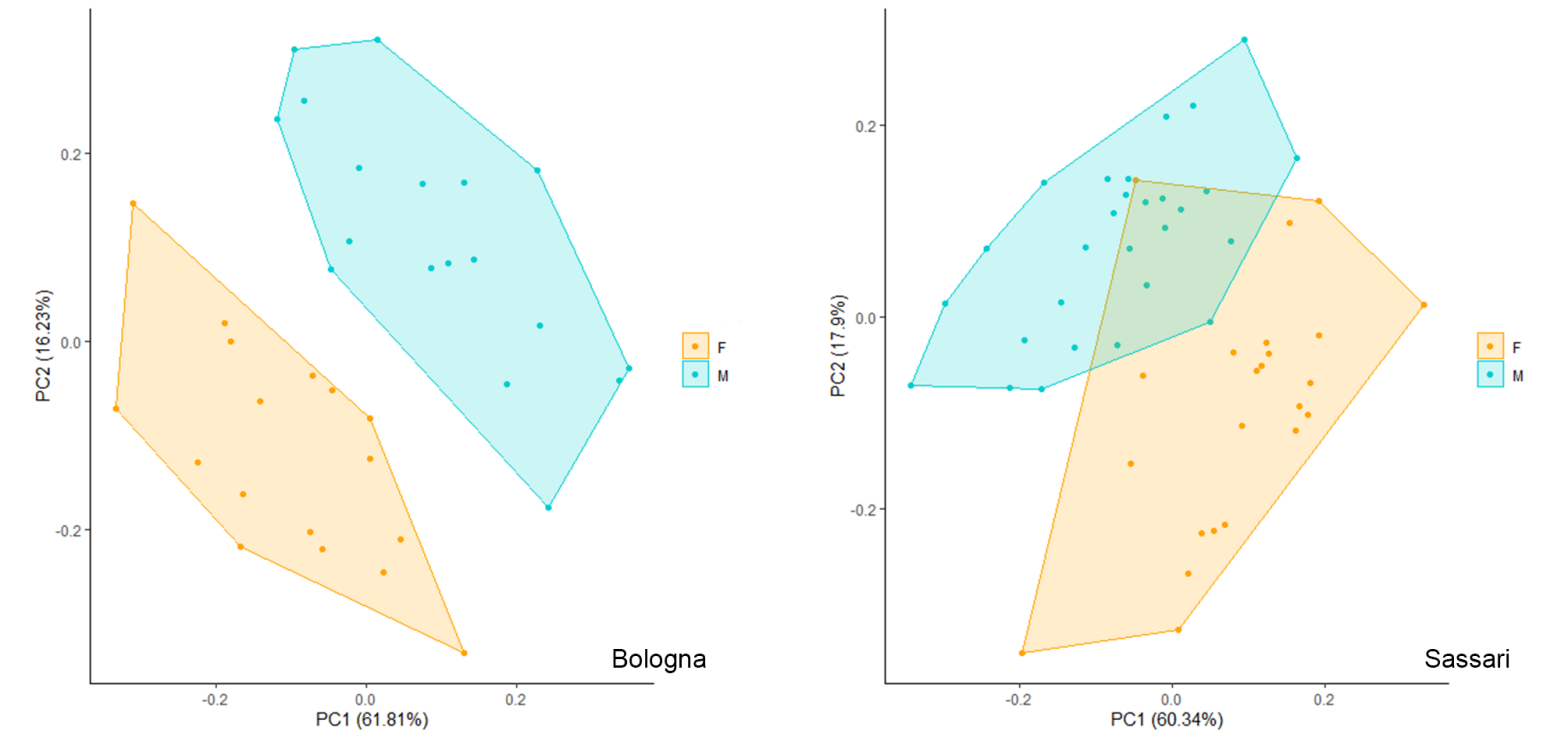
PCA plots showing the variability expressed by the two sexes along PC1 and PC2 for Bologna (on the left) and Sassari (on the right).

Principal Component Analysis (PCA) was carried out for Bologna and Sassari sample separately (Fig. 3) using all resulting dimorphic variables. Measurements with unacceptable ICC values or exhibiting asymmetry were excluded from PCA and following analysis. In the Bologna sample, PC1 (61.81%) and PC2 (16.23%) clearly show a separation between sexes mainly driven by coxal bone maximum height (M01), post-acetabular ischium length (ISMM), and ischium length (ISM) along PC1, and pubis length (PUM), greater sciatic notch height (M15.1), and acetabular pubic length (M17.a) along PC2 (Tab. S2 and Fig. S1). The first two PCs (PC1 60.34% and PC2 17.90% of the total variance) relative to the Sassari sample show an overlap between sexes, although a tendency through separation is appreciable along PC1 mainly driven by coxal bone maximum height (M01), and along PC2 mainly driven by greater sciatic notch height (M15.1) (Tab. S2 and Fig. S1).

The stepwise discriminant analysis selected different sets of variables for Bologna (*N* = 10) and Sassari (*n* = 8) samples with each variable with a high significant score (*p* < 0.0001), meaning that each one has a high discriminant power (Table 7). Accuracy of classification is high for Bologna (100%) with both all dimorphic variables and stepwise selected variables, whereas for Sassari the accuracy decreased at 91.2% in both cases (Table 8).

**Table 7:** Stepwise discriminant function of pelvic measurements for Bologna and Sassari.

Step	Variables	Wilks lambda	F-ratio	Significance
Bologna				
1	M22	0.368	72.026	0.000
2	PUM	0.251	61.012	0.000
3	M01	0.190	56.753	0.000
4	M17.a	0.168	48.334	0.000
5	SS	0.155	41.341	0.000
6	M15.1	0.148	35.486	0.000
7	ISMM	0.139	31.729	0.000
8	M14.1	0.132	28.669	0.000
9	M15.a	0.128	25.632	0.000
10	M18	0.125	23.204	0.000
Sassari				
1	M14.1	0.479	59.709	0.000
2	PUM	0.419	37.385	0.000
3	M01	0.304	40.453	0.000
4	M15.1	0.293	31.339	0.000
5	M21	0.283	25.864	0.000
6	M15.a	0.272	22.306	0.000
7	M20	0.268	19.107	0.000
8	SS	0.265	16.642	0.000

**Table 8:** Accuracy (%) and cross-validation (CV) classification for Bologna and Sassari.

Variables	Total		Males		Females	
	CV	%	CV	%	CV	%
Bologna						
12 measurements	44/44	100.0	25/25	100.0	19/19	100.0
Stepwise selected measurements	44/44	100.0	25/25	100.0	19/19	100.0
Sassari						
8 measurements	52/57	91.2	29/32	90.6	23/25	92.0
Stepwise selected measurements	52/57	91.2	29/32	90.6	23/25	92.0

## Discussion

This study aimed at contributing to the long-standing research investigation on sexual dimorphism in the hip bone, particularly exploring the differences in two skeletal Italian samples, Bologna and Sassari. A good reproducibility of the utilized 19 measurements of the hip bone (Table 3) and the consistent absence of asymmetry (Table 4) allow easy and indifferent use of both sides avoiding loss of information in the case of suboptimal hip preservation.

### Exploring the hip bone sexual dimorphism within and between Bologna and Sassari groups

Regarding the differences between sexes within each sample, all the longitudinal measurements show sexual dimorphism (height of the hip - M01 and pubic symphysis - M18; length of the obturator foramen - M20; length of ischium - ISM and of post-acetabular ischium - ISMM; maximum diameter of the acetabulum - M22; acetabular-ischium length - M15.a). Also, greater sciatic notch length (M15.1), cotylo-sciatic breadth (M14.1), pubis length (PUM), and spino-sciatic length (SS) are highly dimorphic (Table 5). Most of these measurements seem to reflect bigger hip and body size in males, as already observed^20,21,38–44^. Particularly, the diameter of the acetabulum (M22) and the measurements that include its dimension (ISM and ISMM) reflect the bigger femoral head in males than in females, accordingly to other studies that have found the acetabulum dimension and the ischium length amongst the most dimorphic measurements^20,21,44–47^.

In addition, the ischiopubic complex, as expressed by acetabular pubic length (PUM), pubic symphysis heigh (M18), acetabular ischium length (M15.a), ischium length (ISM), post-acetabular ischium length (ISMM), and obturator foramen length (M20), reveals shape differences between sexes within each population, depicting a longer pubis and shorter ischium in females than males, likely accounting for childbearing adaptations of the pelvis^39,45,46,48–50^.

These results are in line with other studies that have considered samples coming from the Mediterranean area. In detail, coxal bone maximum height (M01), acetabular ischial length (M15.a), and maximum diameter of acetabulum (M22) differ significantly between sexes in Greek, Spanish, Portuguese and other Italian populations^20,21,44,46^. Considering the Apulian sample, the obturator foramen length (M20), the post-acetabular ischium length (ISMM), and the spino-sciatic length (SS) differ significantly between sexes^20^. However, interpopulation differences are also observed: the general hip antero-posterior dimensions (coxal bone depth - M04; iliac breadth maximum - M12; breadth of iliac fossa - M13; spino-auricular length - SA; acetabular-symphyseal breadth - M14) for both the samples here analyzed, the acetabular pubic length (M17.a) for Sassari and obturator foramen breadth (M21) for Bologna, are not dimorphic, while they highly discriminate the sex in other Mediterranean populations^20,21,44,46^.

In the studied samples, longitudinal metric traits demonstrate a higher degree of dimorphism than antero-posterior dimensions, resulting in a comparable dimorphic shape between the two italian populations. However, although similarly sexually dimorphic, Bologna samples are larger in size compared to those from Sassari. Indeed, significative metric differences between the two samples are observed as both Bologna males and females showed relatively greater length dimensions than Sassari ones (Tables 5 and 6; Fig. 2). This reflects the general bigger body size in the Bologna population compared to the Sassari one (height: 162.15–163.94 cm in the Sardinian population; 167.52–169.31 cm in Emilia-Romagna region of individuals born in 1927^35^). The general small body size of the Sardinian people, quite typical in the islands, is observed in the past and modern population as well. A few variations of the stature have been observed with different methodologies on skeletal Sardinian remains starting from the recent Neolithic (4000 − 3200 BC) to the modern age (15th -19th c.), where the individuals showed about the same values with about 10 cm of differences between sexes (162–165 cm males; 151–156 cm females)^51^. This may account for the high heritability coefficient of the stature (80%^52^), considering the peculiar genetic background of the Sardinian population that differs from other Mediterranean and Italian populations^53^. In addition, it has been observed that the coefficient of heritability for all the skeletal lengths (e.g., height, sitting height) tends to be higher compared to other metrics (e.g., biiliac and biacromial breadths) that likely express more environmental influence during growth^54^. This reinforces the statement that also the differences in the hip longitudinal metrics observed in the samples are under strong genetic control.

Even though, environmental factors, such as harsh environmental and nutritional constraints, or a physiological/epigenetic response to extreme deprivation, strongly affect the adult size in Sardinian population^36^. On the other hand, some variables that characterize the antero-posterior and medio-lateral size of the coxal bone (e.g., M04, M15.1, M 17.a, PUM, SS, SA) show lower differences between Sassari and Bologna samples in both the sexes (Tables 4 and 5; Fig. 2).

### Comparing the extent of sexual dimorphism between these samples

Considering the second aim of the study, the extent of the sexual dimorphism within Bologna and Sassari, respectively, observed through the PCA (Fig. 2), shows a certain degree of overlap between Sassari males and females, as opposed to a clear separation between sexes in the Bologna sample. This result is also evident with the linear discriminant analysis (Table 8), that indicates a lower discriminant power of the variables we utilized for the Sassari sample in comparison to the Bologna ones. Indeed, considering both all and stepwise selected variables, we obtained excellent percentages of accuracy (100%) for Bologna, and lower, although good, percentages of accuracy (91.2%) for Sassari. Although the percentages of accuracy can be considered overall satisfactory for both samples, Sassari shows a relatively lower accuracy compared to that obtained by some of the discriminant functions reported in the literature for the coxal bone for the Mediterranean area: 90–96% of correct classification for Spanish populations^55^, 79.1–93.5% for Greek populations^46^, 98.6–100% for Portuguese populations^44^, 100% for Apulian populations^20^. Even so, the accuracy percentages obtained by metric analyses are well above those obtained when using only morphological approaches, previously tested on the same samples of Bologna and Sassari^56^. Stepwise discriminant analysis (Table 7) highlights sex prediction power for different sets of variables which among those revealed as significantly dimorphic by ANOVA or Kruskal Wallis analyses (Table 5). The different variable selection between Bologna and Sassari sample highlights region-specific metric characterization that could probably be related to differences in body size.

Thus, the different sexual dimorphic extent of the Sassari sample compared to Bologna could be due to the lower body size, but also to other factors, above mentioned, namely the genetic ones may have played a role to explain this behavior. The role of a long genetic isolation of the Sardinian population has been recently confirmed to explain its small body size. The ‘island rule’ effects may also be a contributing factor to the smaller size of Sardinian population, that is also observable in many other mammal species that live in the islands^37^. Regarding the genetic signature, a greater degree of genomic variability has been highlighted in the Italian sample compared to other European countries, but there are also genetic differences between Sardinia and Italian mainland, attesting the existence of a genetic barrier between those two areas^30,31,53^. Short or long-range mobility within the Italian peninsula and from/to the surrounding countries may explain this picture. Indeed, since the prehistory, those migrating phenomena progressively shaped the genetic background of the continental Italy over time, showing for the modern Italian population a high degree of genetic differentiation^33^. On the contrary, the genetic history of Sardinia, from the arrival of the Neolithic farmers from the Middle Eastern Europe and until the beginning of the first millennium BC, is characterized by no (or minimal) evidence for gene flow from distinct ancestries until the late Bronze Age^57^.

The smaller size together with the relatively lesser degree of hip sexual differences in the Sardinia sample compared to the Bologna one suggests a relationship between body size and sexual dimorphism, showing lower average stature and dimension of the acetabular metrics, which can be considered as a proxy of body size. Firstly, among humans, males show higher body size than females. Indeed, the male-biased size dimorphic populations, in which males are larger than females, approximately comprise 45% species of mammals, and male mammals are the largest sex (average male/female mass ratio 1.184), at least 10% larger than females. In addition, the Rensch’s rule^58,59^ stated that, also among mammals, the more dimorphic species tend to be larger, with sexual size dimorphism tending to scale with body size. Primates, including humans, fit into the pattern of increasing sexual size dimorphism with increasing body size^24^, while sexual size dimorphism is lacking in smaller mammals^25^. In addition, even though there is no general agreement^11^, at the species level the differential allometric growth trajectories of males and females lead to intensified pelvic dimorphism as a consequence of high body size dimorphism, under the influence of growth hormones. Thus, the patterns of sexual dimorphism in humans show that populations with high body size dimorphism should also display high pelvic dimorphism^60^. Finally, ecological conditions, climate, and especially the temperature affect the human body mass average, resulting in significantly smaller mass average during periods of climatic warming as compared to cooler cycles^61,62^. Among mammals, those inhabiting cooler climates are generally larger than their close relatives from warmer climates^63^. Thus, in a microevolutionary perspective, the lower size and lesser degree of sexual dimorphism in the Sassari sample could account for that general pattern, considering the geographic and ecological features of the Sardinia. In addition, considering its peculiar genetic background, the well-known ‘island rule’ that brings to a reduction of the body size that is a pervasive phenomenon across vertebrates^37^, may be invoked to explain the features we observed.

Finally, socio-economic factors may also contribute relevantly to the different body features and size between the two samples, and consequently the different hip bone size, stemming from different growth trajectories. At the onset of the Italian Kingdom, about 70% of the population worked in agriculture, but marked differences in access to resources were observed between the North and South (with islands), with dramatic consequences in the lifestyle, social, cultural, and economic situation^64^. The demographic composition and occupation-at-death of the individuals of Bologna, dated to 19th -20th centuries, indicate a quite low social status, and the presence of infectious diseases is also attested^27,65^. Nevertheless, in that period, the modernization and urbanization processes occurring in whole northern Italy, played a role in generating a diffuse improvement of living conditions. The city of Bologna, in fact, witnessed major urbanistic renovations and public décor interventions, such as the openings of wide urban arteries and the inaugurations of modern gas and lighting systems, initiating the major growth of urban population from its province^66,67^. On the other hand, the Sassari province, despite its urban center also played a role as urbanization pole in northern Sardinia and was involved in renovations of its structures and infrastructures during the last decades of 19th century, was characterized by worst socio-economic and health conditions compared to the mainland. The endemic presence of malaria was also widespread, favored by the marshy fields of alluvial territory^68^.

## Conclusion

In summary, this study highlights regional differences within the Italian population in hip bone metrics, widely used for sex determination.

Our study confirms the well-known role of the hip bone in accounting for the human sexual dimorphism, furthering our understanding of its relationship to body-size in the samples analysed. Indeed, the hip bone size is highly sexually dimorphic in both the Bologna and Sassari specimen, especially for the longitudinal dimensions, whereas the antero-posterior metrics do not to express significative sexual differences, likely accounting for stronger genetic vs. environmental influence.

Concurrently, the extent of the hip bone sexual dimorphism is larger in Bologna than in Sassari, likely reflecting the larger body size in the former sample. Thus, our results indicate a relationship between pelvic and body size, underlying a relevant dimorphic effect due to the size due to the genetic factors.

These microevolutionary processes on the body size and the extent of sexual dimorphism differences, stress the relevance of this local variations for accurate diagnostic criteria.

Further tests comparing the estimation of the body mass (e.g., femoral head) and stature (e.g., length of the femur) for Bologna and Sassari individuals with their relative extent of sexual dimorphism are needed. It is also important to note that the reduction criteria and selection of complete cases have decreased the sample size for discriminant analysis, which may ultimately affect the results. Additionally, the samples are regionally biased, as this skeletal sample may not fully represent the broader genetic and environmental variability of these two Italian populations. Future analyses should also include other documented collections from other Sardinian and mainland Italian territories in further analyses.

## Materials and methods

### The samples

Skeletal remains from the DHOCs of the University of Bologna^27,28,69^ are well preserved, and individuals with bone disease were not included in this study. The sample is composed of 70 individuals (36 males and 34 females) coming from the Certosa Cemetery of Bologna (Emilia Romagna, northern Italy) and 70 individuals (36 males and 34 females) from the Sassari cemetery (Sardinia). All individuals composing the sample are adults, as dimorphism reaches full expression after puberty under the influence of sex hormones. It is important to remind that the correspondence between the cemetery archive, the historical information about the Certosa cemetery^70^, and information on sex and age-at-death of Bologna individuals has been thoroughly checked^27^. Additionally, for the Bologna samples, the most common morphological methods for sex determination have been applied to test their reliability on skull and coxal bone, with the last element showing an accuracy ranging between 90% and 99%^56^. For the Sardinian collection, demographic data come from lists filled out when this collection entered in the first years of the 20th century at the University of Bologna.

### Statistical analyses

 A metric analysis of the coxal bone was conducted to assess whether the standard methods could be equally valid for both groups with equal accuracy. Standard equipment and techniques were used to record 19 coxal bone measurements (Table 1; Fig. 1)^71^. In cases where a missing portion of the coxal bone prevented the registration of one or more metric variables, individual metric variables were excluded from the analysis on a case-by-case basis. All measurements were repeated by the same observer on 40 randomly selected individuals two months after the initial registration, and by a second observer on 10 randomly selected individuals to test the reliability of the variables. Inter- and intra-observer errors were evaluated using the intraclass correlation coefficient (ICC) with the R package “IRR”. The paired t-test was used to assess significant differences for side (asymmetry). Descriptive statistics (mean, standard deviation and range) are provided for Sassari and Bologna and the distribution of each variable was visualized through boxplots. The normal distribution of each variable in our database was verified using the Shapiro–Wilk test. Subsequently, based on the normality distribution of the sample, Analysis of Variance (ANOVA) or Kruskal-Wallis test was conducted for Bologna and Sassari to identify significant differences between sexes for each variable. Moreover, ANOVA or Kruskal-Wallis was used to identify differences between males and females in each of the two Italian samples, respectively. Principal Component Analysis (PCA) was performed to highlight patterns of metric variation between sexes within each sample. Multivariate normality tests (using R package “MVN”) were conducted for all resulting dimorphic variables as a group, while measurements with unacceptable ICC values or displaying asymmetry were excluded from further analysis. Stepwise discriminant analysis was carried out to determine the best discriminant variables for Sassari and Bologna, respectively, using the Wilk’s Lambda criterion^72^. Linear discriminant analysis was then performed on the overall resulting discriminant variables from ANOVA or Kruskal-Wallis tests, as well as on the stepwise selected measurements for Sassari and Bologna to assess the degree of accuracy for each group. This includes cross-validation and accuracy for separate and combined males and females of each group. An accuracy of ≥ 80% was considered an accurate classification^73^.

All statistical analyses were conducted in R v.4.2.3.

## Electronic supplementary material

Below is the link to the electronic supplementary material.

Supplementary Material 1

## Data Availability

All data needed to evaluate the conclusions in the paper are present in the manuscript and/or the Supplementary Information. The datasets and codes used for generating results of this paper are stored at the AMS Acta Institutional Research Repository of Alma Mater Studiorum University of Bologna, reachable through the following link: https://doi.org/10.6092/unibo/amsacta/7886.
