# Supplementary material for: The extent of the hip bone sexual dimorphism in two Italian coeval modern skeletal samples
Source: Sci Rep. 2025 Jan 19;15:2439. doi: 10.1038/s41598-025-86197-3 (PMC11743771; doi:10.1038/s41598-025-86197-3)
Supplement: Supplementary file 1 — Supplementary Material 1 [file 41598_2025_86197_MOESM1_ESM.pdf]

Table S1. The Shapiro–Wilk test.

Variables	Bologna males		Bologna females		Sassari males		Sassari females	
	W	*p*-value	W	*p*-value	W	*p*-value	W	*p*-value
M01	0.975	0.619	0.982	0.857	0.974	0.545	0.972	0.519
M04	0.945	0.173	0.981	0.939	0.979	0.785	0.942	0.163
M12	0.942	0.169	0.967	0.473	0.955	0.219	0.957	0.252
M13	0.970	0.546	0.969	0.483	0.975	0.579	0.965	0.338
M14	0.972	0.578	0.964	0.580	0.973	0.547	0.969	0.573
M 14.1	0.959	0.210	0.968	0.412	0.931	0.027	0.895	0.003
M15.1	0.951	0.134	0.978	0.697	0.964	0.296	0.974	0.569
M15.a	0.950	0.115	0.955	0.177	0.958	0.192	0.985	0.903
M17.a	0.985	0.923	0.962	0.554	0.978	0.704	0.968	0.568
M18	0.931	0.059	0.920	0.075	0.970	0.467	0.954	0.314
M20	0.931	0.031	0.962	0.302	0.965	0.331	0.976	0.651
M21	0.920	0.015	0.973	0.629	0.968	0.419	0.975	0.620
M22	0.955	0.174	0.921	0.017	0.983	0.852	0.919	0.015
PUM	0.969	0.494	0.955	0.414	0.962	0.281	0.953	0.273
ISM	0.978	0.701	0.961	0.263	0.951	0.114	0.952	0.140
SPU-dx	0.932	0.037	0.974	0.586	0.940	0.052	0.880	0.001
SPU-sx	0.923	0.026	0.923	0.022	0.950	0.099	0.969	0.431
ISMM	0.949	0.107	0.956	0.187	0.993	0.999	0.931	0.033
SS	0.981	0.809	0.965	0.345	0.943	0.061	0.957	0.217
SA	0.980	0.764	0.982	0.836	0.925	0.017	0.985	0.919

Table S2. Loadings of variables for PC1 and PC2.

	Bologna		Sassari	
Variables	PC1	PC2	PC1	PC2
M01	0.531	0.076	0.567	0.207
M04	0.333	0.574	0.295	0.644
M12	0.283	0.158	0.316	0.195
M13	0.121	0.143	0.162	0.219
M14	0.250	0.205	0.226	0.185
M 14.1	0.165	0.125	0.099	0.113
M15.1	0.025	0.254	0.015	0.322
M15.a	0.227	0.111	0.266	0.120
M17.a	0.081	0.313	0.087	0.240
M18	0.129	0.134	0.123	0.105
M20	0.090	0.013	0.111	0.062
M21	0.017	0.103	0.006	0.040
M22	0.174	0.185	0.178	0.123
PUM	0.066	0.367	0.094	0.242
ISM	0.351	0.261	0.304	0.213
ISMM	0.326	0.288	0.317	0.260
SS	0.233	0.174	0.226	0.137
SA	0.144	0.092	0.121	0.104

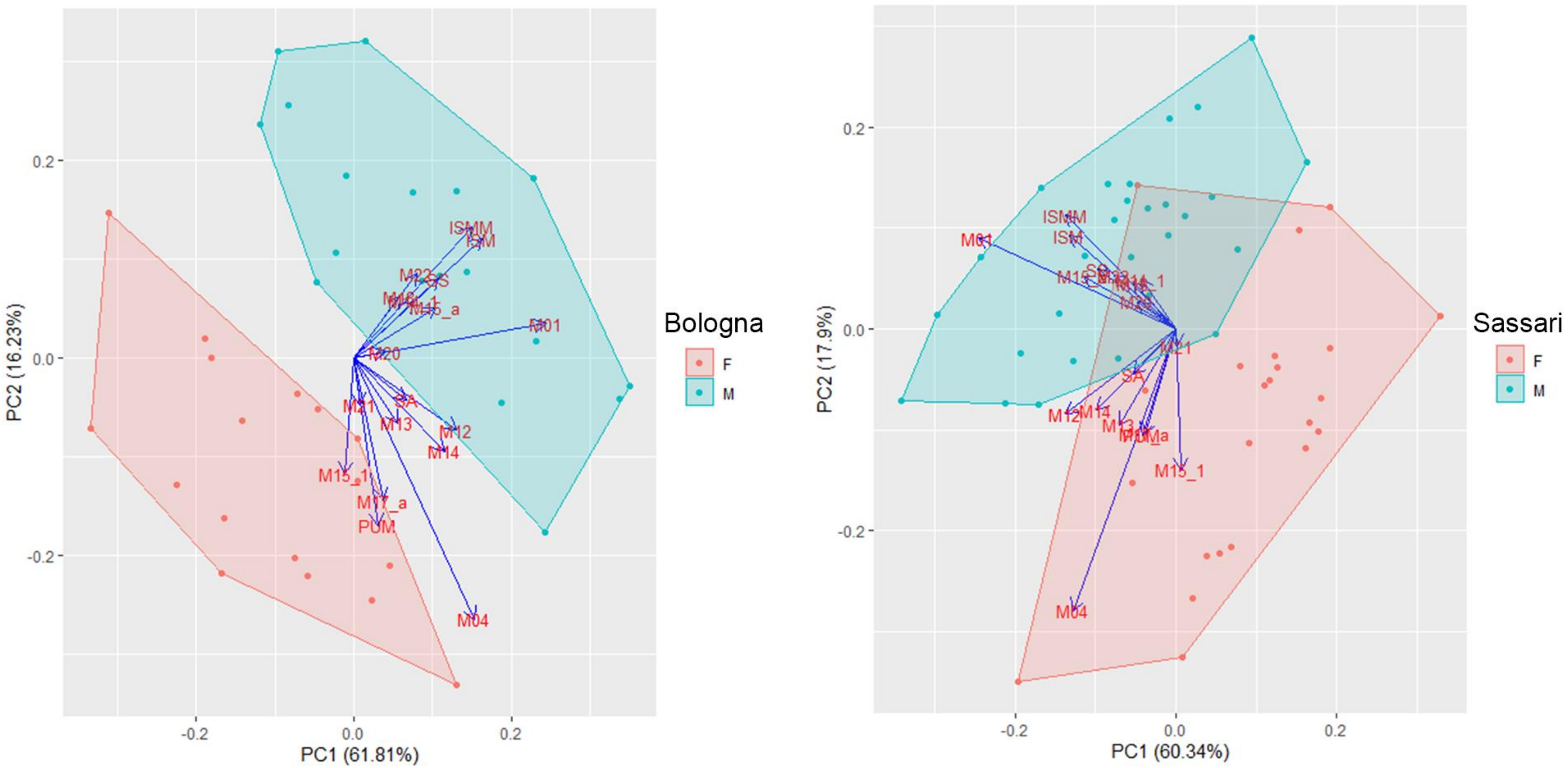

Figure S1. PCA plots illustrating the variability between the two sexes along PC1 and PC2 for Bologna (on the left) and Sassari (on the right). The loadings are also depicted, highlighting the contribution of each variable to the principal components.
